# Reproducibility of Telomere Length Assessment - An International Collaborative Study

**DOI:** 10.1093/ije/dyv171

**Published:** 2015-09-24

**Authors:** Carmen M. Martin-Ruiz, Duncan Baird, Laureline Roger, Petra Boukamp, Damir Krunic, Richard Cawthon, Martin M Dokter, Pim van der Harst, Sofie Bekaert, Tim de Meyer, Goran Roos, Ulrika Svenson, Veryan Codd, Nilesh J Samani, Liane McGlynn, Paul G Shiels, Karen A Pooley, Alison M Dunning, Rachel Cooper, Andrew Wong, Andrew Kingston, Thomas von Zglinicki

International Journal of Epidemiology 2014, doi: 10.1093/ije/dyu191

Key Messages

Rankings are similar if different laboratories measure telomere lengths in the same samples.

TLR values for Labs 3 and 4 in round 2 as shown in Tab. 2 were not calculated from the set of raw values shown in suppl. Tab. S2, and this error was propagated through the following analyses. In addition, in suppl. Tab. S3, Pearson correlations were shown instead of Spearman’s correlation coefficients. Results based on the set of raw data shown in suppl. Tab S2 are provided below.

We correct the following statements (corrections underlined):

*Results:* Absolute results from different laboratories differed widely and could thus not be compared directly, but most rankings of relative telomere lengths were highly correlated (correlation coefficients 0.25 −0.99).

**Table 2.** TLR as measured in the participating labs and inter-lab CVs in round 1 (to) and round 2 (bottom)

Sample	Round 1												
	Lab 1	Lab 2	Lab 3	Lab 4	Lab5	Lab6	Lab7	Lab8	Lab9	CV for All Labs	CV for qPCR Labs	CV for qPCR triplets (median)	CV for South & STELA
	South	South	STELA	qPCR	qPCR	qPCR	qPCR	qPCR	qPCR				
A	1.19	1.07	1.35	1.13	1.06	1.23	1.44	0.91	1.10	13.78	15.60	14.75	11.67
B	1.15	1.34	1.28	0.65	1.18	1.14	1.21	1.34	1.16	17.89	21.44	19.23	7.68
C	1.91	1.61	1.85	1.51	1.72	1.53	2.35	1.55	1.78	15.17	18.40	13.66	8.92
D	1.08	1.26	1.07	0.59	0.66	0.83	1.13	0.83	0.63	27.45	25.75	18.78	9.38
E	0.63	0.87		0.44	0.22	0.36	0.79	0.13	0.31	57.53	61.59	52.56	22.86
F	0.63	0.79	0.79	0.39	0.19	0.28	0.46	0.39	0.14	53.46	40.54	42.92	12.80
G ^a^	1.00	1.00	1.00	1.00	1.00	1.00	1.00	1.00	1.00				
H	0.64	0.68	0.75		0.17	0.31	0.33	0.31	0.13	57.93	36.87	41.46	7.79
I	0.91	1.11	0.94	1.30	1.52	1.10	1.80	1.39	1.79	25.44	18.65	16.93	10.86
J	0.90	0.95	0.94	0.88	0.86	0.83	1.15	0.89	0.89	10.21	12.83	9.74	2.68

Sample ^b^	Round 2														
	Lab 1	Lab 2	Lab 3	Lab 4	Lab5	Lab6	Lab7	Lab8	Lab9	Lab 10	Lab 10-2	CV for All Labs	CV for qPCR Labs	CV for qPCR triplets (median)	CV for South & STELA
	South	South	STELA	qPCR	qPCR	qPCR	qPCR	qPCR	qPCR	qPCR	qPCR				
B	1.39	1.37	1.51	0.84	1.13	1.06	1.43	1.20	0.99	0.85	0.98	20.62	18.48	16.69	5.54
C	1.52	1.54		1.54	1.73	1.54	1.50	1.64	1.73	1.93	1.55	11.06	12.32	8.93	6.31
C	1.56	1.53	1.76	1.78	1.55	1.59	1.61	1.53	1.65	1.63	0.99				
K	0.99	1.05	1.12	0.68	0.58	0.74	1.04	0.84	0.50	0.73	0.74	24.89	22.15	19.87	6.24
L		0.99	1.08	0.83	0.42	0.60	0.65	0.60	0.65	0.45	0.73	30.74	22.21	19.99	6.53
G	0.94	0.95	0.94	1.02	0.97	1.03	0.94	1.00	1.01	1.11	1.33	7.93	8.64	4.64	3.25
G ^a^	1.00	1.00	1.00	1.00	1.00	1.00	1.00	1.00	1.00	1.00	1.00				
H	0.57	0.69	0.75	0.38	0.19		0.31	0.31	0.11	0.12	0.19	62.27	39.89	37.06	13.19
H	0.56	0.71	0.77	0.38	0.21	0.27	0.28	0.29	0.11	0.13	0.21				
I	0.87	0.99	0.99	1.28	1.48	1.25	1.32	1.37	1.61	1.44	1.38	18.17	8.48	7.26	7.67
											**Median**	**22.76**	**20.05**	**17.86**	**7.74**

TLR, telomere length ratio; CVs, coefficients of variation.

^a^ All TLR values were calculated as the ratio of the estimated telomere length for a particular sample, divided by the estimated telomere length for sample G.

^b^ The second round of measurements was designed to enable inter-batch comparison and included 5 repeat samples from the first round (B, C, G, H, I), of which samples C, G and H were duplicated (for intra-batch comparison). CVs for qPCR labs were higher than those for Southern/STELA labs (p = 0.000, paired t-test).

**Figure 1.**
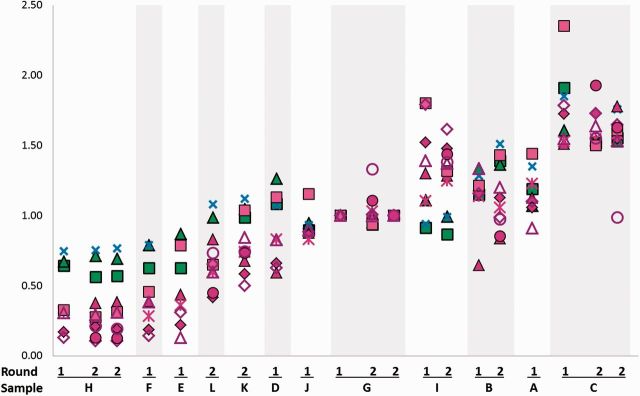
Telomere length ratios (TLRs) by laboratory, round and sample. TLRs are normalized to sample G, first round. Symbols indicate laboratories and techniques. Green indicates SOUTH, blue indicates STELA and pink symbols indicate qPCR. ▪ Lab 1 South; ▴ Lab 2 South; ✗ Lab 3 STELA; ▴ Lab 4 qPCR; ♦ Lab 5 qPCR; * Lab 6 qPCR; ▪ Lab 7 qPCR; Δ Lab 8 qPCR; ♦ Lab 9 qPCR; • Lab 10 qPCR duplex; ○ Lab 10-2 qPCR monoplex.

**Figure 2.**
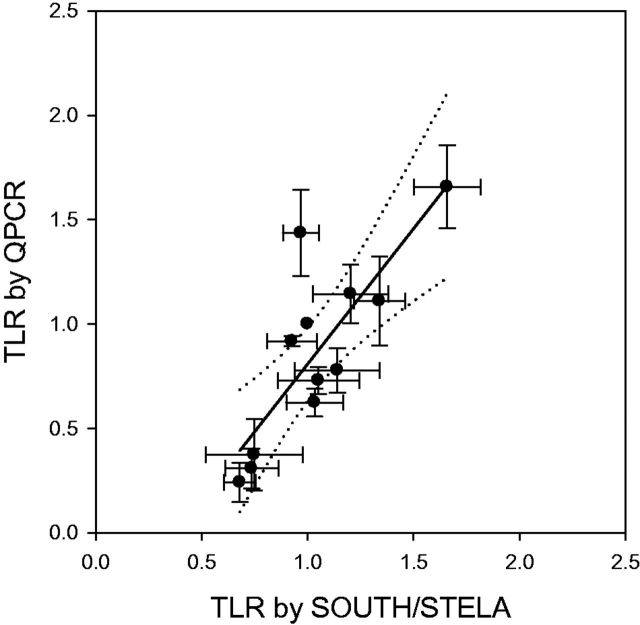


**Figure 3.**
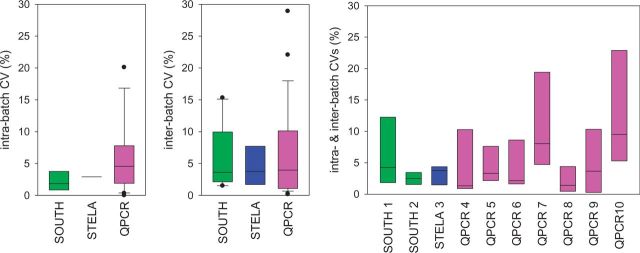
(a–c).

**Table 3.** Intra-batch CVs per laboratory

Sample	Lab 1	Lab 2	Lab 3	Lab 4	Lab5	Lab6	Lab7	Lab8	Lab9	Lab 10	Lab 10-2
name	South	South	STELA	qPCR	qPCR	qPCR	qPCR	qPCR	qPCR	qPCR	qPCR
C	1.702	0.178		10.294	7.799	1.903	4.771	4.566	3.354	11.934	31.299
G	4.614	3.481	4.374	1.331	2.162	2.156	4.721	0.324	0.470	7.095	20.089
H	1.083	2.007	1.489	1.304	7.018		8.985	3.861	0.000	2.404	6.264

**Table 4.** Inter-batch CVs per laboratory

Sample	Lab 1	Lab 2	Lab 3	Lab 4	Lab 5	Lab 6	Lab 7	Lab 8	Lab 9
name	Tech 1	Tech 1	Tech 2	Tech 3	Tech 3	Tech 3	Tech 3	Tech 3	Tech 3
B	13.388	1.499	11.627	17.989	3.046	5.215	11.522	7.431	11.314
C	15.305	3.368	3.772	6.497	3.564	1.652	28.906	1.709	3.973
G	2.270	1.719	2.154	0.669	1.073	1.086	2.322	0.162	0.235
H	8.813	2.980	1.259		11.650	8.925	7.144	0.850	13.671
I	3.877	7.991	3.755	0.897	2.175	8.620	22.052	1.093	7.395

**Suppl. Table S3.** Spearman’s rank correlation coefficients between participating laboratories

Round 1	Lab 1 South	Lab 2 South	Lab 3 STELA	Lab 4 qPCR	Lab 5 qPCR	Lab 6 qPCR	Lab 7 qPCR	Lab 8 qPCR	Lab 9 qPCR
Lab 1 South	1.000								
Lab 2 South	0.855	1.000							
Lab 3 STELA	0.983	0.867	1.000						
Lab 4 qPCR	0.650	0.600	0.524	1.000					
Lab 5 qPCR	0.770	0.855	0.750	0.900	1.000				
Lab 6 qPCR	0.879	0.818	0.867	0.867	0.939	1.000			
Lab 7 qPCR	0.770	0.842	0.750	0.883	0.952	0.915	1.000		
Lab 8 qPCR	0.770	0.806	0.700	0.867	0.952	0.867	0.867	1.000	
Lab 9 qPCR	0.709	0.818	0.667	0.883	0.988	0.903	0.939	0.939	1.000

Round 2	Lab 1 South	Lab 2 South	Lab 3 STELA	Lab 4 qPCR	Lab 5 qPCR	Lab 6 qPCR	Lab 7 qPCR	Lab 8 qPCR	Lab 9 qPCR	Lab 10 qPCR	Lab 10-2 qPCR
Lab 1 South	1.000										
Lab 2 South	0.933	1.000									
Lab 3 STELA	0.929	0.950	1.000								
Lab 4 qPCR	0.700	0.685	0.483	1.000							
Lab 5 qPCR	0.783	0.855	0.667	0.927	1.000						
Lab 6 qPCR	0.690	0.733	0.476	0.933	0.967	1.000					
Lab 7 qPCR	0.883	0.927	0.817	0.806	0.927	0.917	1.000				
Lab 8 qPCR	0.800	0.842	0.650	0.915	0.988	0.967	0.939	1.000			
Lab 9 qPCR	0.695	0.693	0.477	0.985	0.936	0.917	0.802	0.936	1.000		
Lab 10 qPCR	0.683	0.733	0.500	0.976	0.952	0.933	0.830	0.939	0.985	1.000	
Lab 10-2 qPCR	0.450	0.576	0.250	0.867	0.855	0.750	0.661	0.842	0.912	0.927	1.000

**Suppl. Table S4.** z-scored results from all participating laboratories in round 1 (top) and 2 (bottom) and inter-laboratory variation in z scores (as standard deviation) between all laboratories and separated by technique

Lab 1	Lab 2	Lab 3	Lab 4	Lab 5	Lab 6	Lab 7	Lab 8	Lab 9	Lab 10	lab 10-2	SD	SD	SD South/
South	South	STELA	qPCR	qPCR	qPCR	qPCR	qPCR	qPCR	qPCR	qPCR	all	qPCR	STELA
round 1													
0.453	−0.012	0.751	0.480	0.306	0.703	0.663	−0.033	0.319			0.288	0.271	0.385
0.344	0.928	0.540	−0.666	0.527	0.492	0.239	0.876	0.417			0.463	0.524	0.296
2.414	1.894	2.280	1.395	1.538	1.419	2.370	1.330	1.484			0.453	0.389	0.270
0.165	0.670	−0.094	−0.795	−0.426	−0.242	0.083	−0.210	−0.491			0.426	0.298	0.390
−1.075	−0.727		−1.172	−1.245	−1.358	−0.561	−1.699	−1.030			0.357	0.379	0.246
−1.073	−1.004	−0.958	−1.280	−1.303	−1.541	−1.176	−1.159	−1.312			0.181	0.138	0.058
−0.061	−0.264	−0.320	0.177	0.201	0.165	−0.162	0.161	0.147			0.209	0.137	0.136
−1.035	−1.416	−1.092		−1.333	−1.474	−1.423	−1.327	−1.337			0.158	0.067	0.205
−0.295	0.129	−0.506	0.891	1.166	0.413	1.338	0.998	1.496			0.723	0.382	0.324
−0.338	−0.457	−0.519	−0.117	−0.054	−0.236	0.124	−0.066	−0.049			0.213	0.117	0.092
round 2													

0.998	1.029	1.242	−0.217	0.435	0.300	0.643	0.591	0.124	−0.143	0.140	0.483	0.317	0.133
1.349	1.635		1.454	1.549	1.455	0.778	1.521	1.386	1.624	1.397	0.398	0.414	0.244
1.449	1.622	1.987	2.030	1.215	1.554	0.974	1.302	1.250	1.131	0.169			
−0.093	−0.098	0.043	−0.592	−0.572	−0.455	−0.095	−0.177	−0.705	−0.336	−0.365	0.247	0.210	0.080
	−0.313	−0.073	−0.231	−0.880	−0.794	−0.817	−0.704	−0.444	−0.803	−0.387	0.289	0.242	0.170
−0.233	−0.434	−0.503	0.222	0.146	0.239	−0.283	0.151	0.158	0.273	0.919	0.310	0.245	0.157
−0.061	−0.264	−0.320	0.177	0.201	0.165	−0.162	0.161	0.147	0.100	0.195			
−1.228	−1.349	−1.077	−1.313	−1.295		−1.446	−1.317	−1.376	−1.336	−1.566	0.138	0.104	0.123
−1.252	−1.278	−1.028	−1.296	−1.258	−1.561	−1.517	−1.352	−1.376	−1.329	−1.527			
−0.428	−0.292	−0.351	0.853	1.081	0.754	0.429	0.953	1.193	0.819	1.024	0.615	0.235	0.068
										**median**	0.310	0.245	0.170

**Table 5.** Test results

Analysis		original value in the paper		corrected value	
Spearman’s rank correlation coefficients (abstract and results p4 1 ^st^ para)		Range: 0.63 −0.99		Range: 0.25 −0.99	
Paired T-test CVs (SB+STELA) vs CVs qPCR (Results p.4 2 ^nd^ para)		p = 0.001		p = 1.8x10 ^−7^	
Linear regression of LTRs South/STELA vs qPCR (p6 1 ^st^ para)		Offset: −0.55 ± 0.32		Offset: −0.49 ± 0.32	
		Slope: 1.38 ± 0.30		Slope: 1.30 ± 0.30	
Intra-batch CV values (Table 3, p6 2 ^nd^ para)	Differences between labs	Labs 1 to 10-1 ANOVA, p = 0.299		Labs 1 to 10.1 ANOVA, p = 0.299	
				Labs 1 to 10-2 Kruskal-Wallis, p = 0.089	
	Median intra-batch CVs per technique	1.86% (SB); 2.83% (STELA); 4.57% (qPCR)		1.86% (SB); 2.93% (STELA); 4.57% (qPCR)	
	Differences between techniques (Kruskal-Wallis)	p = 0.161		p = 0.201	
	Differences between techniques with SOUTH and STELA combined (Mann-Whitney)	p = 0.075		p = 0.082	

Analysis		original value in the paper		corrected value	
Inter-batch CV values (Table 4, p7)	Differences between labs (Kruskal-Wallis)	p = 0.195		p = 0.190	
	Median inter-batch CVs per technique	3.62% (SB); 4.78% (STELA); 4.65% (qPCR)		3.62% (SB); 3.76% (STELA); 3.97% (qPCR)	
	Differences between techniques (Kruskal-Wallis)	p = 0.840		p = 0.842	
Intra- & Inter-batch CV values combined (p7, 2 ^nd^ para)	Differences between labs (Kruskal-Wallis)	p = 0.060		p = 0.052	
GLM (p8)	Null hypothesis of equal variance between all groups	F = 1.650; p = 0.096		F = 1.998; p = 0.036	
		Partial eta-squared coefficients:		Partial eta-squared coefficients:	
		LAB	.013	LAB	.014
		TECHN	.000	TECHN	.000
		LAB * TECHN	.000	LAB * TECHN	.000

